# Genome-Wide Identification and Characterization of *Alternative Oxidase* (*AOX*) Genes in Foxtail Millet (*Setaria italica*): Insights into Their Abiotic Stress Response

**DOI:** 10.3390/plants13182565

**Published:** 2024-09-12

**Authors:** Hui Zhang, Yidan Luo, Yujing Wang, Juan Zhao, Yueyue Wang, Yajun Li, Yihao Pu, Xingchun Wang, Xuemei Ren, Bo Zhao

**Affiliations:** 1 Houji Laboratory in Shanxi Province, College of Life Sciences, Shanxi Agricultural University, Taiyuan 030031, China; huizhang@sxau.edu.cn (H.Z.); 18103579789@163.com (Y.L.); wangyujing0420@163.com (Y.W.); 18049023013@163.com (Y.P.); wxingchun@163.com (X.W.); renxuemei@sxau.edu.cn (X.R.); 2 Department of Basic Sciences, Shanxi Agricultural University, Jinzhong 030801, China; zhaojuan121768@163.com; 3 College of Agriculture, Shanxi Agricultural University, Jinzhong 030801, China; wangyueyue12322@163.com (Y.W.); lyajun0704@163.com (Y.L.)

**Keywords:** foxtail millet, alternative oxidase, abiotic stress, gene expression, haplotype analysis

## Abstract

*Alternative oxidase* (*AOX*) serves as a critical terminal oxidase within the plant respiratory pathway, playing a significant role in cellular responses to various stresses. Foxtail millet (*Setaria italica*), a crop extensively cultivated across Asia, is renowned for its remarkable tolerance to abiotic stresses and minimal requirement for fertilizer. In this study, we conducted a comprehensive genome-wide identification of *AOX* genes in foxtail millet genome, discovering a total of five *SiAOX* genes. Phylogenetic analysis categorized these SiAOX members into two subgroups. Prediction of *cis*-elements within the promoter regions, coupled with co-expression network analysis, intimated that SiAOX proteins are likely involved in the plant’s adaptive response to abiotic stresses. Employing RNA sequencing (RNA-seq) and real-time quantitative PCR (RT-qPCR), we scrutinized the expression patterns of the *SiAOX* genes across a variety of tissues and under multiple abiotic stress conditions. Specifically, our analysis uncovered that *SiAOX1*, *SiAOX2*, *SiAOX4*, and *SiAOX5* display distinct tissue-specific expression profiles. Furthermore, *SiAOX2*, *SiAOX3*, *SiAOX4*, and *SiAOX5* exhibit responsive expression patterns under abiotic stress conditions, with significant differences in expression levels observed between the shoot and root tissues of foxtail millet seedlings. Haplotype analysis of *SiAOX4* and *SiAOX5* revealed that these genes are in linkage disequilibrium, with Hap_2 being the superior haplotype for both, potentially conferring enhanced cold stress tolerance in the cultivar group. These findings suggest that both *SiAOX4* and *SiAOX5* may be targeted for selection in future breeding programs aimed at improving foxtail millet’s resilience to cold stress.

## 1. Introduction

Foxtail millet (*Setaria italica*) is an environmentally friendly crop known for its drought resistance, water-use efficiency, and tolerance to poor soil conditions. It serves as an important strategic reserve for addressing environmental challenges [1,2]. Foxtail millet, along with its wild ancestor, green bristlegrass (*Setaria viridis*), emerged as a model system for investigating stress tolerance and C_4_ photosynthesis in cereals. This shift underscores the growing relevance of uncovering the unique genetic and regulatory mechanisms underpinning their resilience [3].

In plant respiratory metabolism, two electron transport chain pathways are pivotal: the cytochrome pathway and the cyanide-resistant alternative pathway, with the former being the main route [4]. The latter, featuring the alternative oxidase (AOX) at its terminus, operates independently of the cytochrome pathway. AOX facilitates electron transfer from ubiquinone to oxygen, bypassing the proton pump complexes and decoupling ATP synthesis. This cyanide-resistant pathway is particularly significant when plants encounter anions, such as cyanides and azides, that inhibit the cytochrome pathway by binding to cytochrome oxidase, thereby impairing electron transport efficiency and overall respiration [5].

The survival and distribution of plants in natural environments are primarily con-strained by extreme temperatures, drought, and saline soils [6]. These environmental stressors, collectively termed osmotic stresses, result in diminished water availability and typically enhance the synthesis of abscisic acid (ABA) [7]. Elevated ABA levels significantly induce stomatal closure, modulate gene expression, and influence the plant’s adaptive physiological responses [7,8]. A key player in ABA signaling during seed development and in response to various environmental stresses is the ABSCISIC ACID INSENSITIVE3 (ABI3) protein. Cold and heat stress, as well as the exogenous application of abscisic acid, were shown to alter the levels of *GmABI3* in soybean seeds and leaves [9]. Another crucial factor is FUSCA3 (FUS3), a B3 domain transcription factor (TF) that regulates ABA synthesis and interacts with nucleoside diphosphate kinase 1, a component integral to plant cellular processes, development, and stress responses [10]. The role of ABI4 in the regulation of the alternative oxidase gene *AOX1a* was elucidated through the study of *abi4* mutants, which exhibit increased transcript abundance of *AOX1a*, indicating that ABI4 functions to suppress *AtAOX1a* expression. Conversely, the addition of ABA stimulates the expression of *AtAOX1a* [11,12]. The interplay between ABA signaling and *AOX* expression warrants further investigation to fully understand the underlying regulatory mechanisms.

AOX, a member of the diiron-carboxylate protein family, is widely distributed across fungi, protozoa, and higher plants [13]. In 1987, AOX was first isolated from *Sauromatum guttatum* [14]. Hydrogen sulfide, known for its inhibitory effects on cytochrome oxidase, was prevalent at notably high concentrations during the prokaryotic era, predating the emergence of land plants approximately 600 million years ago [15]. This period coincided with a significant increase in atmospheric oxygen levels. It is hypothesized that the early evolution of the AOX was propelled by the unique capabilities of diiron proteins. These proteins were adept at reducing oxygen to water, thereby playing a crucial role in the transition from an anaerobic to an aerobic environment. Notably, this process was not impeded by the presence of sulfide, which suggests a strategic adaptation in the face of environmental challenges [11,15]. Over the course of symbiotic evolution, the AOX enzyme in plant mitochondria likely evolved in tandem with their symbiotic microorganisms, including fungi and protists [16].

Mitochondrial AOX is generally located on the inner membrane of mitochondria and can regulate energy metabolism [17]. AOX is proposed to play essential roles in strong light stress tolerance. Under strong light stress, the activity and expression of *AOX* increase significantly in *Arabidopsis* [5]. Intriguingly, the correlation between strong light-induced metabolic responses and respiratory activities across diverse C_3_ species hints at the AOX pathway’s potential to support stress-related amino acid synthesis, thereby preserving photosynthetic activity [5]. Additionally, when the nuclear-encoded *AOX* gene is knocked out, the delay in chlorophyll accumulation during greening is more pronounced under strong light condition [18].

Moreover, plenty of evidence shows that AOX also functions in balancing reactive oxygen species (ROS), inhibiting the production of free radicals to enhance plants’ resistance to other abiotic stress [17]. Transcriptional upregulation of *AOX* genes or proteins in response to metal toxicity in *Arabidopsis* [19], chilling in peach fruit [20], heat stress in *Pleurotus ostreatus* [21], drought stress in *Solanum lycopersicum* [22], and salt and drought stress in rice [23] are well-documented. Transgenic plants with elevated *AOX* expression levels demonstrated enhanced abiotic stress resistance. For instance, the overexpression of *AOX* genes in tobacco could be relieving aluminum toxicity, through modulating their respiratory capacity and response to oxidative stresses [24]. Enhancing the transcription level of *AtAOX1a* in *Arabidopsis* could improve its adaptability to UV-B radiation through altering the metabolic pathway [25]. Resistance to cold stress was improved by strengthening the expression of some *AOX* genes in winter wheat via reducing the production of reactive oxygen species [26]. The overexpression of *BnaAOX1b* in rapeseed plants resulted in improved germination under osmotic and salt stress compared to ordinary plants [27]. In *Arabidopsis*, *AtAOX1a* and *AtAOX1d* both act to limit oxidative stress during proline catabolism and thereby facilitate plant recovery from osmotic stress [28].

*AOX* is encoded by two nuclear gene subfamilies, *AOX1* and *AOX2*, with dicotyledonous plants possessing both gene subfamilies, while most monocotyledonous plants (except some basal groups) only have the *AOX1* gene [16,17,29,30]. Despite molecular characterizations of the *AOX* family being conducted in several species, including *Arabidopsis* [31], sweet orange [32], rice and barely [33], watermelon [34], moso bamboo [35], and wheat [36], a comprehensive understanding of the *AOX* gene family in foxtail millet still remains elusive. The rapid advancements in third-generation sequencing technology and assembly strategies significantly enhanced the completeness and accuracy of the high-quality reference genome of foxtail millet, thereby empowering us to achieve a more precise identification of gene families [3,37]. In this study, we identified five *SiAOX* genes and performed a comprehensive bioinformatics analysis, including phylogenetic relationships, gene and protein structure, chromosomal localization, and promoter sequence characterization. Additionally, we detailed their tissue-specific and stress-responsive expression patterns. This research provides a robust foundation for unraveling the functional roles of *SiAOX* genes and offers critical insights that can be leveraged to explore and harness genetic resources for enhancing abiotic stress tolerance in foxtail millet.

## 2. Results

### 2.1. Identification and Characterization of AOX Gene Family in Foxtail Millet

**Figure 1:**
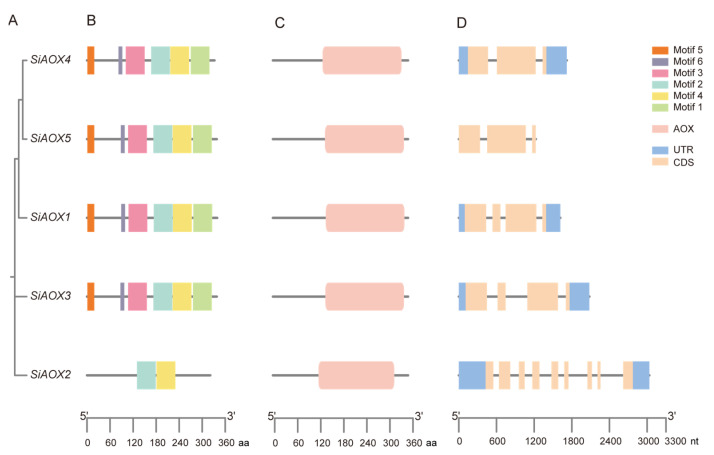
Conserved motifs, functional domain, and gene structure of five SiAOX members in foxtail millet. These sizes could be estimated using the scale at bottom. (**A**) Gene tree. (**B**) Motif patterns. Conserved motifs in the SiAOX peptides are presented by different colored boxes. (**C**) Conserved domain. AOX domain is represented by pink box, other regions of SiAOX peptides are represented by lines. (**D**) Gene structure. Coding sequences (CDS) and untranslated region (UTR) are represented by different colored boxes, and introns are represented by lines.

In the present study, we identified five *AOX* genes within the foxtail millet genome (xiaomi_v1.0) using a combination of BLASTP and HMMER searches. Notably, the *SiAOX* family members emerged as the intersecting entities between the two datasets. The presence of the AOX domain in these proteins was subsequently confirmed using the Pfam database (PF01786). All AOX proteins had the AOX domain (Figure 1), and they were designated based on their chromosomal positions (Figure S1). The five SiAOX proteins showed a range of amino acids with lengths from 321 to 339, molecular weights (MWs) from 37.09 to 37.81 kDa, theoretical isoelectric point (pI) from 5.67 to 8.91, and GRAVY from −0.138 to −0.283 (Table 1). Subcellular localization analysis revealed that SiAOX2 was localized on the plasma membrane, whereas the remaining four proteins are targeted to the mitochondria (Table 1).

### 2.2. Structure Characterization and Three-Dimensional Modeling Analysis of AOX Genes in Foxtail Millet

The structural characteristics of the *SiAOX* gene family were thoroughly examined to gain insights into their potential functions. The exon–intron architecture of the *SiAOX* genes is depicted on the right side of Figure 1, with *SiAOX4* and *SiAOX5* each containing two introns, *SiAOX1* and *SiAOX3* each featuring three introns, and *SiAOX2* possessing a more complex structure with eight introns.

**Figure 2:**
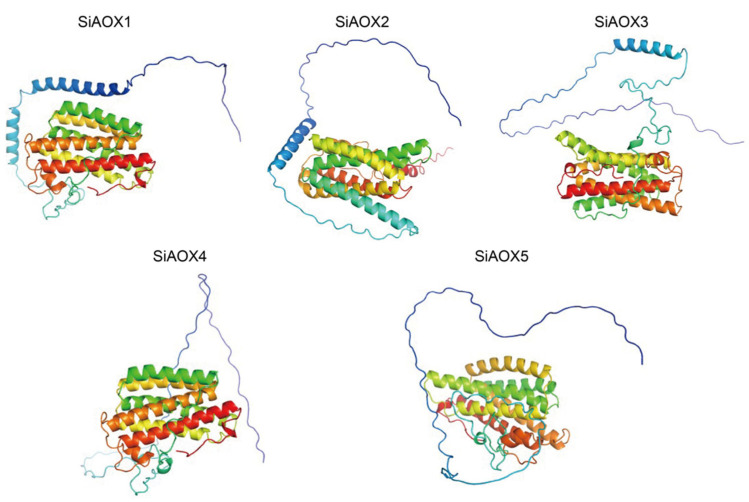
The three-dimensional models of AOX proteins in foxtail millet. All three-dimensional models are constructed using AlaphFold2 v2.3.2 and visualized by Pymol v2.5.5.

**Figure 3:**
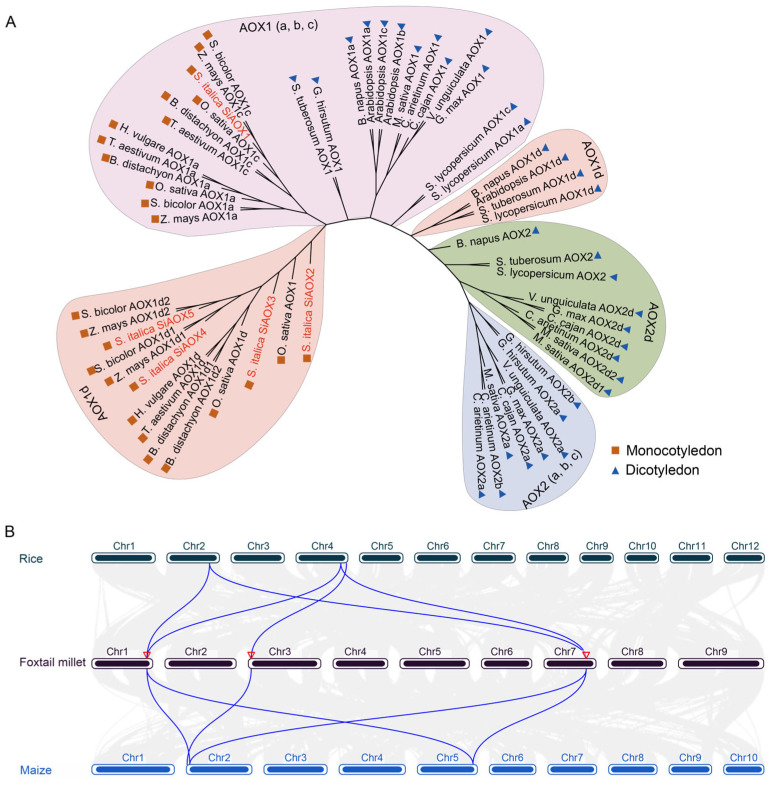
Phylogenetic and collinearity analysis of AOXs in foxtail millet and other species. (**A**) Phylogenetic tree. Phylogenetic tree is constructed by AOX proteins of 17 species, including foxtail millet, rice, *Brachypodium distachyon*, *Triticum aestivum*, *Hordeum vulgare*, maize, sorghum, *Solanum tuberosum*, *Gossypium hirsutum*, *Brassica napus*, *Arabidopsis*, *Medicago sativa*, *Cicer arietinum*, *Cajanus cajan*, *Vigna unguiculata*, *Glycine max*, and *Solanum lycopersicum* with the neighbor-joining (NJ) method using MEGA11. Different colored ellipses represent different evolutionary clades and four clades are labeled with AOX1 (a, b, c), AOX1d, AOX2 (a, b, and c), and AOX2d. AOX proteins in foxtail millet are labeled in red. (**B**) Collinearity of *AOX* genes between foxtail millet, rice, and maize.

The three-dimensional modeling of the SiAOX proteins revealed intricately folded structures, comprised of various secondary structural elements such as alpha-helices, extended strands, beta-turns, and random coils (Table S1, Figure 2). While all SiAOX proteins exhibited similar overall spatial configurations and conserved functional regions, notable variations were observed in the N-terminal peptide sequences (Figure 2). Collectively, the SiAOX proteins that are closely related on the phylogenetic tree also shared similar motif compositions and gene structures (Figure 3A). This observation suggests that members of the same subgroup may fulfill analogous functional roles within the plant’s physiological processes.

### 2.3. Phylogenetic Classification of AOX Proteins in Foxtail Millet

To elucidate the phylogenetic relationships and classify the SiAOX proteins, we constructed a phylogenetic tree using a dataset of AOX proteins from 17 diverse species (Table S2). The neighbor-joining (NJ) method, as implemented in MEGA11 software, was employed for this analysis. The resulting phylogenetic tree delineated the AOX proteins into two distinct subfamilies, namely AOX1 and AOX2. The AOX1 subgroup could be further divided into AOX1 (a, b, and c) and AOX 1d, while AOX2 subgroup could be further divided into AOX2 (a, b, and c) and AOX2d (Figure 3A). The result shows that dicotyledons contained both AOX1 and AOX2 subfamilies, while monocotyledons only had AOX1. This conclusion is consistent with previous research [16,29], and they also mentioned that *Poales* lost the AOX2 subfamily in evolution, which was also verified in our research. According to the phylogenetics tree, SiAOX1 was classified in AOX1c clade, and SiAOX2, SiAOX3, SiAOX4, and SiAOX5 were classified in AOX1d clade. SiAOX proteins showed homology with maize (*Zea mays*), sorghum (*Sorghum bicolor*), and rice (*Oryza sativa*).

### 2.4. Promoter Analysis and Prediction of miRNA Target Sites of AOX Genes in Foxtail Millet

**Figure 4:**
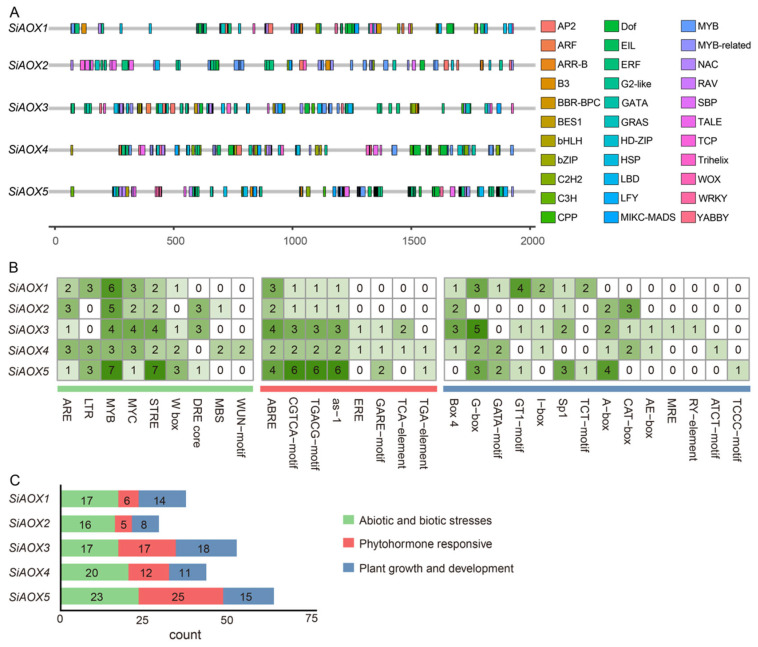
Analysis of *cis*-elements in the *SiAOX* genes promoter regions. (**A**) The distribution of various *cis*-elements in the promoter regions. The different colored blocks represent the different types of *cis*-elements and their locations in upstream 2000 bp of *SiAOX* genes. (**B**) The *cis*-elements in the promoter regions of each *SiAOX* gene. The different colors and numbers in the grid indicate the numbers of different promoter elements in the *SiAOX* genes. Vertical bars with different colors indicate different *cis*-element types. (**C**) Count of three types of *cis*-elements in *SiAOX* genes promoter regions.

To deepen our understanding of the roles played by *cis*-elements in the *SiAOX* genes, an analysis was conducted to identify these elements within the 2000 base pair (bp) upstream sequence from the translation start site (ATG) of each gene, utilizing the plantCARE website A variety of *cis*-elements were identified across all *SiAOX* genes, which could be categorized into three functional groups: those responsive to abiotic and biotic stresses, those involved in phytohormone responsive, and those related to plant growth and development (Figure 4). Notably, the largest proportion of identified *cis*-elements were associated with responses to abiotic and biotic stresses. These included, in descending order of frequency: MYB transcription factor binding sites, stress-responsive elements (STREs), MYC transcription factor binding sites, anaerobic stress elements (AREs), low-temperature responsive elements (LTRs), WRKY binding sites (W boxes), dehydration-responsive elements (DRE cores), MYB binding sites implicated in drought inducibility (MBSs), and wound-responsive elements (WUN motifs). Additionally, phytohormone-responsive elements, including those responding to abscisic acid (ABREs), methyl jasmonate (JeJA-responsiveness motifs, TGACG and CGTCA), and as-1 (SA and oxidative responsiveness elements), were found in all *SiAOX* promoter regions. For the group associated with plant growth and development, several tissue-specific light response elements were identified, including Box 4, Sp1, GATA motifs, CAT boxes, G-boxes, and I-boxes (Figure 4). These findings suggest that the *SiAOX* genes may play a role in the plant’s response to various abiotic and biotic stresses.

MicroRNAs (miRNAs), which are non-coding single-stranded RNA molecules, are known to regulate gene expression post-transcriptionally [38]. The results of predicted miRNA target sites show that (Table S3), except SiAOX3, a total of 18 miRNA target sites were predicted across four *SiAOX* genes. These sites are targeted by 18 different types of miRNAs. Among the 16 predicted interactions, 61.11% (11 out of 18) miRNAs silenced the target *SiAOX* genes by cleavage of their transcripts.

### 2.5. Colinearity and Selective Pressure Analysis of AOXs in Foxtail Millet

To investigate the homologous gene pairs and the selective pressures acting on the *SiAOX* genes, we conducted a collinearity analysis among rice, foxtail millet, and maize. The collinearity analysis revealed significant collinearity between the chromosome segments harboring the *SiAOX1*, *SiAOX2*, and *SiAOX3* genes and their respective homologous regions on rice chromosomes 2 and 4, and maize chromosomes 2 and 5 (Figure 3B). In contrast, no collinearity was observed between the chromosome segments containing the *SiAOX4* and *SiAOX5* genes and the corresponding regions with *AOX* genes in rice and maize.

To assess the selective pressures on the *AOX* gene pairs, we employed the ratio of non-synonymous to synonymous substitutions (*Ka*/*Ks*). The *SiAOX* gene pairs were selected based on their closest evolutionary relationship as determined by phylogenetic analysis or their collinearity. The findings indicate that all *SiAOX* gene pairs were under purifying selection, with *Ka*/*Ks* values ranging from 0.1460 to 0.4646 (Table 2).

### 2.6. Expression Patterns of AOX Genes in Different Tissues in Foxtail Millet by RNA-seq

**Figure 5:**
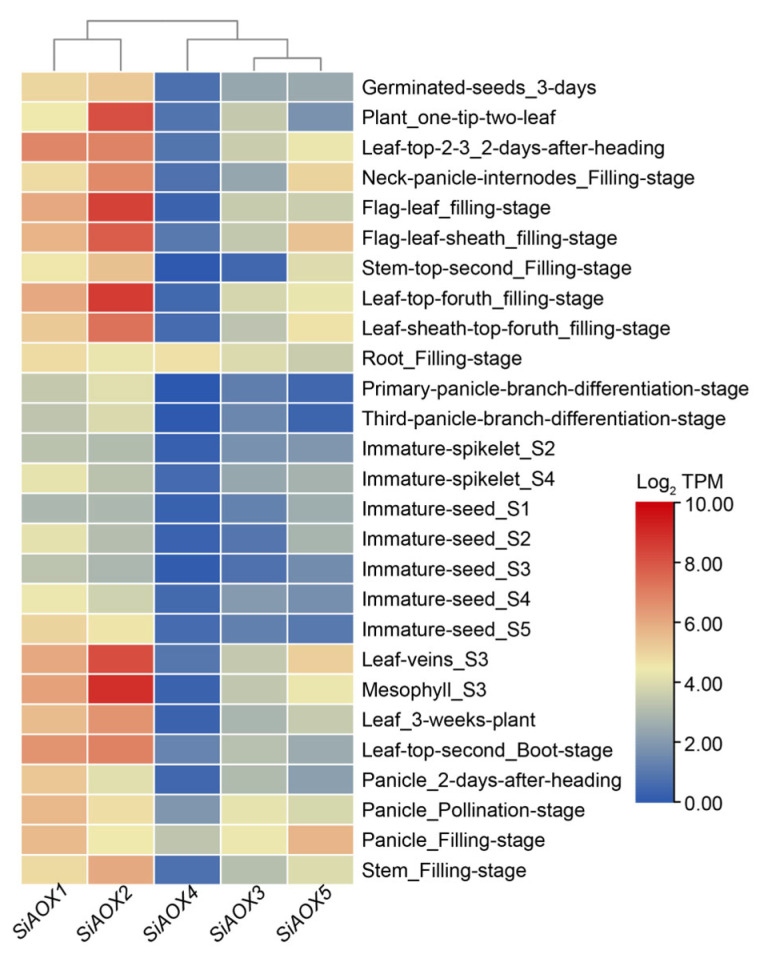
The spatiotemporal expression patterns of *SiAOX* genes in multiple tissues during the whole growth period in foxtail millet. The expression matrices (TPM values) of five *SiAOX* genes in 27 important tissues of foxtail millet during the whole growth period are retrieved from the foxtail millet multi-omics database (MDSi). The data presented were calculated using the log_2_TPM method. The visualization is achieved by TBtools II (v2.102), with blue to red representing the amount of expression from low to high.

To elucidate the role of *AOX* genes in the growth and development of foxtail millet, we characterized the expression profiles of these genes in a range of tissues and developmental stages. Our transcriptome analysis revealed tissue-specific expression profiles for the *SiAOX* genes (Figure 5). *SiAOX1* and *SiAOX2* demonstrated pronounced specificity, with high levels of expression observed in leaf tissues throughout multiple developmental stages. Notably, *SiAOX1* reached peak expression in leaves two days post-heading, whereas *SiAOX2* exhibited maximal expression in leaves during the seed filling stage. *SiAOX3* displayed a more ubiquitous expression pattern, being present in most tissues at varying stages, albeit with relatively moderate expression levels. In contrast, *SiAOX4* showed a distinct preference for root tissues, specifically at the filling stage, while *SiAOX5* was selectively expressed in the panicle and flag leaf sheath during the same developmental phase.

### 2.7. Response of SiAOX Genes under Various Abiotic Stress by Quantitative Real-Time PCR (RT-qPCR)

**Figure 6:**
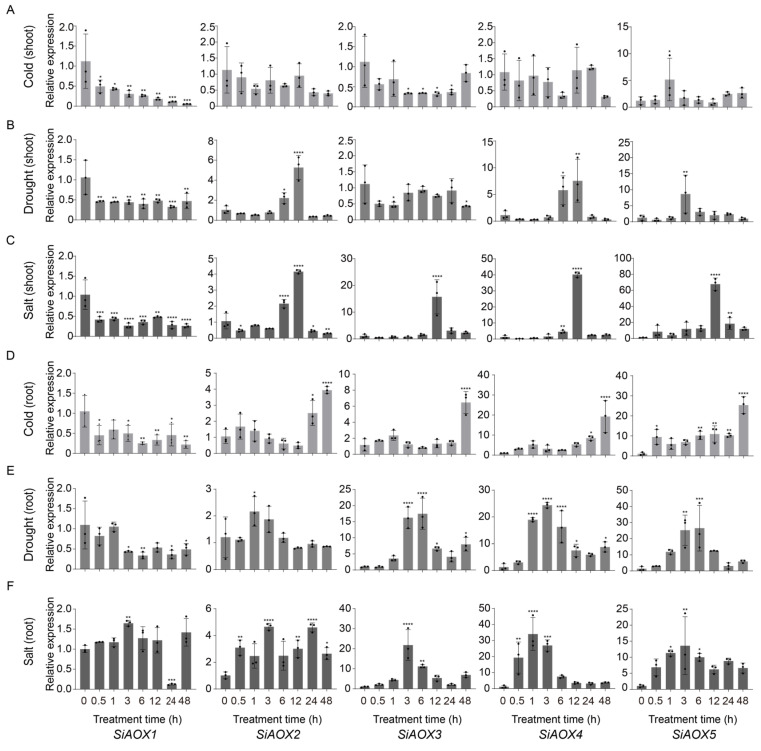
RT-qPCR analysis of *SiAOX* genes under various abiotic stress treatments in shoot and root tissues. (**A**) Expression pattern of *SiAOX* genes in shoot tissue under cold stress. (**B**) Expression pattern of *SiAOX* genes in shoot tissue under drought stress. (**C**) Expression pattern of *SiAOX* genes in shoot tissue under salt stress. (**D**) Expression pattern of *SiAOX* genes in root tissue under cold stress. (**E**) Expression pattern of *SiAOX* genes in root tissue under drought stress. (**F**) Expression pattern of *SiAOX* genes in root tissue under salt stress. The unstressed level (0 h) was used as a control. * Indicates a significant different at *p* < 0.05, ** indicates a significant at *p* < 0.01, *** indicates a significant at *p* < 0.001, and **** indicates a significant at *p* < 0.0001.

To clarify the possible function of *SiAOX* genes in abiotic stresses, we conducted and analyzed RT-qPCR assays on foxtail millet shoots and roots under cold, drought, and salt stress conditions. Under cold stress, *SiAOX5* was significantly upregulated in both tissues, whereas *SiAOX2*, *SiAOX3*, and *SiAOX4* were notably upregulated, specifically in the roots (Figure 6A,D). Their expression peaked at 1 h post-stress in shoots and at 48 h in roots, respectively.

For drought stress, *SiAOX4* and *SiAOX5* showed pronounced upregulation in both tissues, with *SiAOX2* exhibiting a distinct pattern of induction in shoots only, and *SiAOX3* being specifically induced in roots (Figure 6B,E). The expression of these drought-responsive genes followed a biphasic pattern, increasing initially and then decreasing, with the roots showing an earlier response than shoots.

Under salt stress, the *SiAOX* gene family was significantly upregulated in both tissues, with the exception of *SiAOX1* (Figure 6C,F). The salt-responsive *SiAOX* members exhibited an initial upregulation followed by a subsequent downregulation, with the root tissue responding earlier than the shoots.

Comparing the responses across the three abiotic stresses, the *SiAOX* genes were most markedly affected by salt stress, followed by drought and cold stress. The *SiAOX* genes in roots exhibited an earlier response to salt and drought stresses than shoots, with significant upregulation observed at 1 h post-treatment. In contrast, shoots showed a significant increase at 3 h post-treatment. During cold stress, the shoots responded earlier than roots, with root expression levels continuing to rise up to 48 h after treatment. These findings indicate that *SiAOX* genes, with the exception of *SiAOX1*, play crucial roles in the abiotic stress response of foxtail millet.

### 2.8. Co-Expression Network and Haplotype Variations Analysis of AOX Genes in Foxtail Millet

To further decipher the roles of the *SiAOX* gene family members, we examined the co-expression network associated with these genes, derived from the network of differentially expressed genes (DEGs) in foxtail millet seedlings under cold stress conditions, as previously constructed in our study [39].

**Figure 7:**
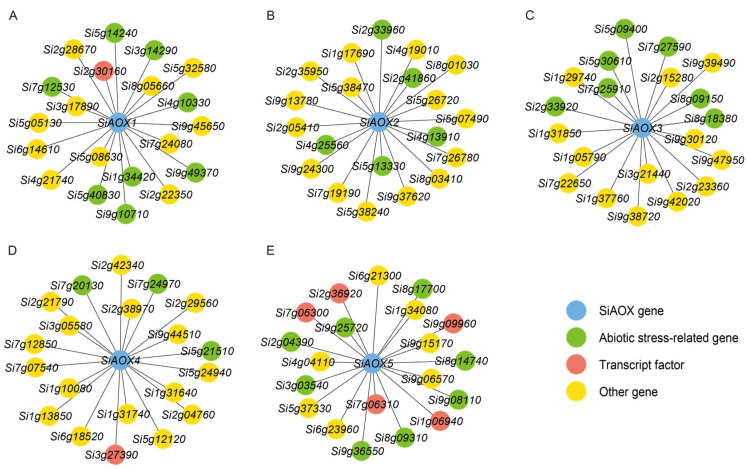
Co-expression network of five *SiAOX* genes in foxtail millet. (**A**) *SiAOX1*. (**B**) *SiAOX2*. (**C**) *SiAOX3*. (**D**) *SiAOX4*. (**E**) *SiAOX5*. The blue circle indicates the core gene that is both belongs to the network and *SiAOX* genes family, and green circle indicates the reported abiotic stress-related genes, the red circle indicates the annotated transcription factors, the yellow circle indicates other genes.

We then annotated and visualized the top 20 DEGs with the highest connectivity within the *SiAOX* co-expression network. The analysis revealed a significant presence of abiotic stress-related and transcription factor genes within each *SiAOX* co-expression network (Figure 7). Notably, the *SiAOX5* network included four transcription factors and eight genes related to abiotic stress. Similarly, the *SiAOX1* and *SiAOX4* networks each contained a transcription factor and eight abiotic stress-related genes, while the *SiAOX3* network comprised seven abiotic stress-related genes, and the *SiAOX2* network contained five. These results suggest that *SiAOX* genes may play a role in the response to abiotic stresses.

**Figure 8:**
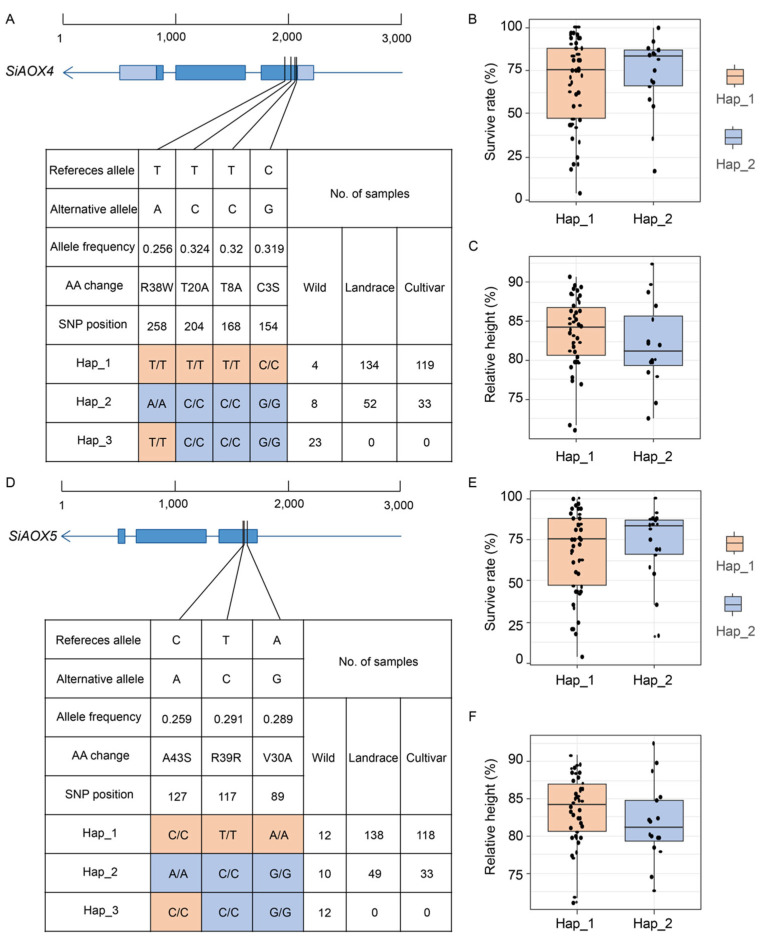
Haplotype analysis of *SiAOX4* and *SiAOX5* genes in foxtail millet. (**A**,**D**) SNPs identified for haplotype analysis of *SiAOX4* and *SiAOX5*, respectively. Haplotype information of foxtail millet resources are provided in Tables S4 and S5 for SiAOX4 and SiAOX5, respectively. Survive rate of *SiAOX4* and *SiAOX5* after cold stress treatment are provided in Table S6 and Table S7, respectively. (**B**,**E**) The survive rate of Hap_1 and Hap_2 after cold stress in *SiAOX4*, *SiAOX5*, respectively. (**C**,**F**) The relative height of Hap_1 and Hap_2 after cold stress in *SiAOX4*, *SiAOX5*, respectively. Relative height of *SiAOX4* and *SiAOX5* after cold stress treatment are provided in Table S8 and Table S9, respectively.

To explore the function of *AOX* gene upon cold stress in seedlings, we conducted a haplotype analysis of the *AOX* genes in foxtail millet. Three haplotypes were identified for *SiAOX4* (Figure 8A), with Hap_1 being the most prevalent (68.9% in all materials), followed by Hap_2 (24.9%) and Hap_3 (6.2%). Hap_1 was most abundant in landraces (52.1%), then in cultivars (46.3%), and least in wild types (1.6%). Hap_2 was most prevalent in landraces (55.9%), followed by cultivars (35.5%), and least in wild types (8.6%). Hap_3 was exclusive to wild types (100%). These findings indicate that *SiAOX4* may be a domesticated gene, with Hap_3 representing the ancestral genotype. Hap_2 was identified as the superior haplotype, associated with a higher survival rate and reduced relative height following cold stress (Figure 8B,C).

*SiAOX5* was also classified into three haplotypes, with Hap_1 being the most common (72.1% in foxtail millet materials), followed by Hap_2 (24.7%) and Hap_3 (3.2%). The distribution trends for *SiAOX5* haplotypes mirrored those of *SiAOX4*, with Hap_1 being the most numerous, Hap_2 the next, and Hap_3 being the rarest and found only in wild types (Figure 8D). This suggests that *SiAOX5* is also a domestication-related gene, with Hap_3 as the ancestral genotype. Hap_2 emerged as the superior haplotype, linked to enhanced survival rate and lower relative height post-cold stress (Figure 8E,F).

**Figure 9:**
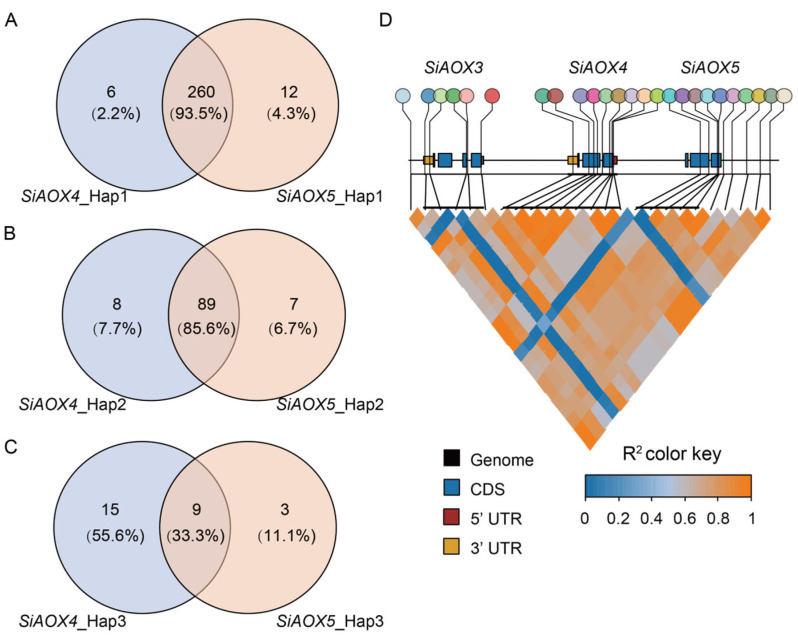
Combination haplotype and LD analysis of the region surrounding *SiAOX4* and *SiAOX5* genes. (**A**–**C**) Venn of materials between *SiAOX4* and *SiAOX5* gene of Hap_1, Hap_2, and Hap_3, respectively. (**D**) LD analysis of *SiAOX3*, *SiAOX4*, and *SiAOX5* genes.

Given the similar performance conferred by different *SiAOX4* and *SiAOX5* alleles following cold stress, we conducted a more detailed analysis of the haplotype distribution across all foxtail millet resources, as well as loci linkage disequilibrium (LD). The results show that Hap_1 of *SiAOX4* and *SiAOX5* shared 93.5% of the material, Hap_2 shared 85.8%, and Hap_3 shared 33.3% (Figure 9A–C). LD results also indicate strong linkage disequilibrium between *SiAOX3*, *SiAOX4*, and *SiAOX5* (Figure 9D), suggesting that their superior haplotypes may be inherited together to the next generation, conferring together to cold tolerance.

## 3. Discussion

### 3.1. SiAOX Genes and Their Classify in Foxtail Millet Genome

The *AOX* gene family is a critical group in organisms, with its encoded proteins playing a central role in cellular energy metabolism and respiratory functions. In plants, the *AOX* gene family is indispensable for regulating growth and development, energy metabolism, and resistance to environmental stresses [36,38,40]. The *AOX* gene family were identified across numerous species, such as *Triticum aestivum* [36], *Phyllostachys edulis* [35], *Citrullus lanatus* [34], *Ipomoea batatas* (L.) Lam. [41], maize, rice, *Arabidopsis*, *Brachypodium distachyon*, *Solanum lycopersicum* [38], *Solanum tuberosum*, sorghum, and *Glycine max* [42]. The number of *AOX* genes varies significantly, from a single gene in *Citrullus lanatus* to 17 in *Triticum aestivum*, highlighting a high level of diversity. In this study, five *AOX* genes were identified in the foxtail millet genome (Figure 1).

Phylogenetic analysis in this study categorized AOX gene family members from 17 species into four subgroups (Figure 3A). The division of AOX proteins from monocotyledonous and dicotyledonous plants into different subgroups on the phylogenetic tree suggests that AOX proteins likely underwent differentiation in various groups, leading to the formation of their respective unique gene families during the early stages of angiosperm evolution. In foxtail millet, the five SiAOX proteins were divided into two subgroups, all of the SiAOXs were classified in the AOX1 subfamily (Figure 3A). Based on the phylogenetic tree, SiAOX1 was categorized under the AOX1c clade, whereas SiAOX2–5 was all grouped together within the AOX1d clade. This conclusion aligns with prior studies [16,29], which confirms the evolutionary loss of the AOX2 subfamily in *Poales* lineage.

### 3.2. SiAOX Genes May Play a Crucial Role in the Response to Abiotic Stresses in Foxtail Millet

During the growth and development of plants, the expression of *AOX* genes is induced by various biotic and abiotic stresses [29,33]. These stresses can trigger *AOX* gene expression, thereby regulating plant respiration to adapt to changes in the external environment [15,20,34]. Under low-temperature stress, AOX can maintain the operation of the tricarboxylic acid cycle, providing plants with necessary energy and stabilizing their growth rate [35,36]. *AOX1* and *AOX2* exist in dicotyledons, but only *AOX1* in monocotyledons [16,29]. *AOX1* expression rises with stress and mitochondrial dysfunction, regulated by mitochondrial retrograde signals [17,43]. Initially thought to maintain housekeeping roles, recent studies show *AOX2* genes also respond to stresses [44,45].

Analysis of the promoter regions of *SiAOX* genes revealed 31 *cis*-acting elements, including those related to abiotic and biotic stresses, plant phytohormone responses, and plant growth and development. This suggests that *SiAOX* may play a crucial role in stress and hormone stress responses, as well as in plant growth and development (Figure 4). As it was reported, in *Arabidopsis* and various other species, the expression of *AOX1A* is intimately linked to mitochondrial dysfunction, with a notable tendency to elevate in response to a diverse array of both abiotic and biotic stresses [17,30,46,47]. Increases in AOX activity are often tied to specific tissue development, such as thermogenesis in reproductive tissues of certain plants [48]. Additionally, under P deficiency, plants develop cluster roots that utilize enhanced AOX activity to efficiently produce citrate and acids, facilitating P solubilization. This heightened AOX activity enables rapid TCA cycle turnover without ATP synthesis constraints [49].

miRNAs, a class of endogenous small noncoding RNAs, are known to negatively regulate gene expression and play crucial roles in plant resistance to abiotic stress [50]. For instance, SlMYB15, targeted by *Solanum lycopersicum* miR156e-3p, enhances cold stress tolerance in tomatoes through ABA and ROS signaling pathways [51]. Additionally, LAC2, which negatively regulates lignin deposition, is downregulated by elevated miR397b levels in *Arabidopsis* roots under water-deficit conditions [52]. Predicted miR397 target genes, such as Casein kinase II and L-ascorbate oxidase precursor, were implicated in drought stress responses [53,54]. In our study, *SiAOX5* and *SiAOX2* are identified as potential targets of miR156 and miR397, respectively (Table S3), and they exhibit significant responses to cold, drought, and salt stresses. Further investigation into the responses of these predicted miRNAs to various abiotic stresses, as well as their regulatory effects on the target *SiAOX* genes, will deepen our comprehension of the post-transcriptional regulation of *SiAOX* genes, particularly in the context of abiotic stress adaptations.

In rice, under salt stress for 24 and 48 h, the expression of *OsAOX1a* and *OsAOX1c* was found to be upregulated in leaf tissues, suggesting a potential role in the leaf’s response to salt stress. Conversely, in root tissues, *OsAOX1a*, *OsAOX1c*, and *OsAOX1d* exhibited downregulation, indicating a tissue-specific response to the stressor [33]. In maize, under cold stress for 12 and 24 h, the expression of *ZmAOX1a*, *ZmAOXd1*, and *ZmAOXd2* was significantly upregulated in roots, while *ZmAOX1c* was downregulated. This suggests a differential response of *AOX* genes to temperature stress in maize roots. Under drought stress, *ZmAOXd2* and *ZmAOX1c* were upregulated in roots, and *ZmAOXd2* showed a downtrend in leaves, indicating a dynamic and stress-specific regulation of *AOX* genes. Under saline-alkaline stress for 12 h, *ZmAOX1c*, *ZmAOXd1*, and *ZmAOXd2* were significantly induced in roots, with less pronounced and less consistent changes in leaves [42].

In this study, RT-qPCR analysis showed that most *SiAOX* genes were induced by these stresses, with the exception of *SiAOX1*, which remained low regardless of stress type or tissue. *SiAOX* genes responded most significantly to salt stress, with an initial upregulation followed by downregulation, and roots showing an earlier response than shoots. For drought, *SiAOX2*, *SiAOX4*, and *SiAOX5* were induced in shoots, while *SiAOX3*, *SiAOX4*, and *SiAOX5* were induced in roots, with an initial upregulation followed by downregulation and an earlier root response. Cold stress uniquely induced *SiAOX5* in shoots and *SiAOX2*, *SiAOX3*, *SiAOX4*, and *SiAOX5* in roots, with the shoot response preceding the root. *SiAOX5* was the only member broadly induced by all three stresses in both tissues, with *SiAOX4* also showing induction, except for a minor response in shoots under cold stress.

These observations collectively indicate that there are qualitative and quantitative differences in the expression patterns of various *AOX* genes under abiotic stresses. The distinct regulation of *AOX* genes in different plant species and tissues under various stress conditions underscores the complexity of their roles in stress response and adaptation.

### 3.3. The Cluster Distribution of SiAOX4 and SiAOX5 May Enhance the Foxtail Millet’s Tolerance to Abiotic Stresses

Haplotype analysis indicated that Hap_1 of *SiAOX4* and *SiAOX5* were the predominant haplotypes, occurring at a higher frequency in cultivars and landraces (74.85%/75.74%) compared to Hap_2 (25.15%/24.26%), suggesting that Hap_1 might have been selected during foxtail millet breeding (Figure 8). However, based on the phenotype of foxtail millet seedlings following cold stress treatment, Hap_2 emerges as an excellent cold-tolerant haplotype, indicating its potential for use in future breeding programs (Figure 8).

In prior research on the *AOX* gene family, a pattern of cluster distribution was observed among certain members in maize, *Glycine max*, sorghum, *Solanum tuberosum*, and *Brachypodium distachyon* [42]. Similarly, within the foxtail millet *AOX* family, *SiAOX3*, *SiAOX4*, and *SiAOX5* are found to exhibit this characteristic cluster distribution. No non-synonymous single nucleotide polymorphism (SNP) was detected within the *SiAOX3* genes. Interestingly, the haplotypes of the candidate genes, *SiAOX4* and *SiAOX5*, demonstrate consistency in two key aspects. Firstly, their corresponding haplotypes are predominantly shared across the foxtail millet population (Figure 9A–C). Secondly, the phenotypic responses to cold stress at the seedling stage are consistent among the same haplotypes (Figure 8B,C,E,F). Additionally, LD analysis indicates a strong association among *SiAOX3*, *SiAOX4*, and *SiAOX5*, suggesting that their favorable haplotypes may be co-inherited by subsequent generations, potentially conferring enhanced tolerance to abiotic stresses (Figure 9D).

## 4. Materials and Methods

### 4.1. Plant Growth Condition and Treatment

Seeds of foxtail millet cultivar “Yugu1” were used in the present study. The seeds are sown in a culture dish with ddH_2_O and cultivated in the growth chamber condition (16 h in the daylight at 26 °C and 8 h in the dark at 22 °C, 30,000 Lux light, and ~50% relative humidity). When the seeds germinate and grow to the two-leaf stage, seedlings with consistent growth are selected and transferred to a hydroponic tray. After fourteen days of growth, the treatments were processed. For cold stress, seedlings were transferred to a growth chamber set to 4 °C. For drought and salt stresses, 20% PEG6000 and 200 mmol/L NaCl + 25 mmol/L Na_2_CO_3_ were filled in a hydroponic tray with Hoagland’s solution, respectively. The shoot and root of seedlings were collected, respectively, and frozen in liquid nitrogen at different time points (0, 0.5, 1, 3, 6, 12, 24, and 48 h) after treatment, and stored at −80 °C.

To investigate the change in *SiAOX* gene expression in foxtail millet under cold, drought, and salt treatment, total RNA was extracted from the shoot and root of foxtail millet with fourteen days of cultivate hydroponically using Total RNA Extract Reagent and RNA Extraction solution (Beijing Coolaber Technology Co., Ltd., Beijing, China), according to the manufacturer’s instructions. All RNA isolation for gene expression was conducted in triplicate for each sample.

### 4.2. Genome-Wide Identification and Characterization of AOX Gene Family in Foxtail Millet

Foxtail millet genome (xiaomi_v1.0) was retrieved from MDSi database (http://foxtail-millet.biocloud.net/home, accessed on 12 December 2023), the accession number is GWHAAZD00000000 in GWH database in the Beijing Institute of Genomics Data Center (https://ngdc.cncb.ac.cn/, accessed on 12 December 2023). Two methods were used to accurately identify all *AOX* genes in foxtail millet (*SiAOX*). First, AOX protein sequences of maize, rice, *Arabidopsis*, *Solanum tuberosum*, *Solanum lycopersicum*, and sorghum were downloaded from the Ensembl Plant database (http://plants.ensembl.org/index.html, accessed on 12 December 2023). Then, AOX protein sequences of six species above were used as seed sequences to align with all *xiaomi* protein sequences by the BLASTP program, filtered with a threshold of E < 1 × 10^−5^. Second, the HMM model (PF01786) of the plant AOX gene family were downloaded from the Pfam database (http://pfam.xfam.org/, accessed on 12 December 2023). After that, Hmmsearch was employed to discern members belonging to the AOX gene family, with a search threshold rigorously set at E < 1 × 10^−5^, ensuring the exclusion of any protein sequences lacking AOX domains. Finally, the *SiAOX* family members were the intersection between the two datasets. The SiAOX peptide sequences were subjected to protein physical and chemical property analysis, including relative molecular weight (MW), theoretical isoelectric point (pI), and grand average of hydropathicity (GRAVY), using the ExPASy proteomics (https://web.expasy.org/protparam/, accessed on 17 December 2023). Subcellular localization of SiAOX proteins was predicted in CELLO_v.2.5 (http://cello.life.nctu.edu.tw, accessed on 17 December 2023).

### 4.3. Chromosomal Localization, Gene Structure, Conserved Domain, Conserved Motif, and Three-Dimensional Modeling Analysis of AOX Genes in Foxtail Millet

The chromosome locations of *SiAOX* genes were obtained from the xiaomi_v1.0 gff3 file. The exon–intron information of *SiAOX* genes was extracted from the xiaomi_v1.0 gff3 file. SiAOX protein sequences were submitted to NCBI CDD (https://www.ncbi.nlm.nih.gov/cdd, accessed on 20 December 2023) databases to confirm the conserved domain. The conserved motifs of SiAOX proteins were detected using the Multiple Expectation Maximization for Motif Elicitation program (MEME; http://meme.nbcr.net/meme/, accessed on 20 December 2023). The analysis was performed with the following parameters: the number of repetitions = any, the maximum number of motifs = 6. Then, the gene structures and conserved motifs were visualized by TBtools-II (v2.102) software. All SiAOX protein sequences were submitted to predict the three-dimensional structure by Alphafold2 (v2.3.2), and the results are visualized by PyMOL (v2.5.5).

### 4.4. Phylogenetic Tree, Selection Pressures and Collinearity Analysis of AOX Proteins in Foxtail Millet

In order to study the evolutionary relationships of AOX proteins, 60 sequences from 17 species, including foxtail millet, rice, *Brachypodium distachyon*, *Triticum aestivum*, *Hordeum vulgare*, maize, sorghum, *Solanum tuberosum*, *Gossypium hirsutum*, *Brassica napus*, *Arabidopsis*, *Medicago sativa*, *Cicer arietinum*, *Cajanus cajan*, *Vigna unguiculata*, *Glycine max*, and *Solanum lycopersicum* were aligned using ClustalW, and the phylogenetic tree was constructed by the neighbor-joining (NJ) method using MEGA11 software (1000 bootstrap replications). The ratios (Ka/Ks) of non-synonymous mutations (Ka) and synonymous mutations (Ks) for paralogous gene pairs were calculated using KaKs_Calculator 2.0. The colinearity analysis of the SiAOX genes in the three species were performed by TBtools-II (v2.102) software.

### 4.5. cis-Regulatory Element Analysis, Prediction of miRNA Target Sites and Tissue Expression Analysis

The upstream 2000 bp of the *SiAOX* genes was considered as a promoter for *cis*-element analysis using PlantCARE (http://bioinformatics.psb.ugent.be/webtools/plantcare/html, accessed on 23 December 2023). All sequences were submitted to the PlantCARE database for *cis*-acting element prediction, and *cis*-acting elements were manually clustered based on the prediction information.

Candidate miRNA targets were identified by blasting the cDNA sequences of *SiAOX* genes to all *Setaria italica* miRNAs we sequenced in our previous study [3].

To examine the expression profiles of the *SiAOX* genes, the RNA-seq data of 27 different tissues from MDSi database were downloaded, and graphs were performed by TBtools-II (v2.102).

### 4.6. RT-qPCR of SiAOX under Cold, Drought and Salt Stresses

Reverse transcription was performed using All-in-One First-Strand Synthesis MasterMix (with dsDNase) (BestEnzymes Biotech Co., Ltd., Lianyungang, China) for Real-time Quantitative Polymerase Chain Reaction (qPCR). F488 SYBR qPCR Mix (Universal) (BestEnzymes Biotech Co., Ltd. Lianyungang, China) was used as a fluorescent dye. The primers of *SiAOX* genes were designed using Primer 5.0 (Table S10), wherein *SiACT1* (*Si5g46030*) was used as the internal reference. RT-qPCR was performed with three biological replicates for each sample. The 2^−ΔΔCT^ method was used to calculate the relative gene expression levels. Statistical analysis of results was reported as means ± SE. Student’s *t*-test or ANOVA determines the significance in SigmaPlot v.11.2.

### 4.7. Co-Expression Network Analysis and Haplotype Analysis of the SiAOX Genes and Association with Traits in Foxtail Millet

In our previous study, the weighted gene co-expression network analysis (WGCNA) was used to analyze RNA-seq data derived from 33 samples, which were collected at different developmental stages and different durations of cold treatment in foxtail millet [39]. This analysis resulted in the construction of 44 co-expression modules. In this study, the module associated with the *SiAOX* genes were visualized using Cytoscape (v3.10.2) and functional annotation on the genes contained within these modules was performed.

All SNPs used for haplotype analysis were obtained from resequencing of foxtail millet resource populations [55]. Haplotype identification and the analysis of phenotypic association and LD were performed by CandiHap [56]. The alleles and corresponding population details of foxtail millet resources are clearly presented in Tables S4 and S5. To ascertain the cold tolerance phenotype of the haplotype, foxtail millet materials were planted and allowed to grow under normal conditions for 14 d, and subsequently subjected to 12 d treatment at 4 °C. The survival count and plant height of both the treated and control groups were meticulously recorded, and the cold tolerance was subsequently calculated utilizing the formula specified. For comprehensive insights, please refer to Tables S6–S9.

Survive rate = count of seedlings after cold stress/count of seedlings before cold stress × 100%

Relative height = (control height − height after cold stress)/control height × 100%

## 5. Conclusions

**Figure 10:**
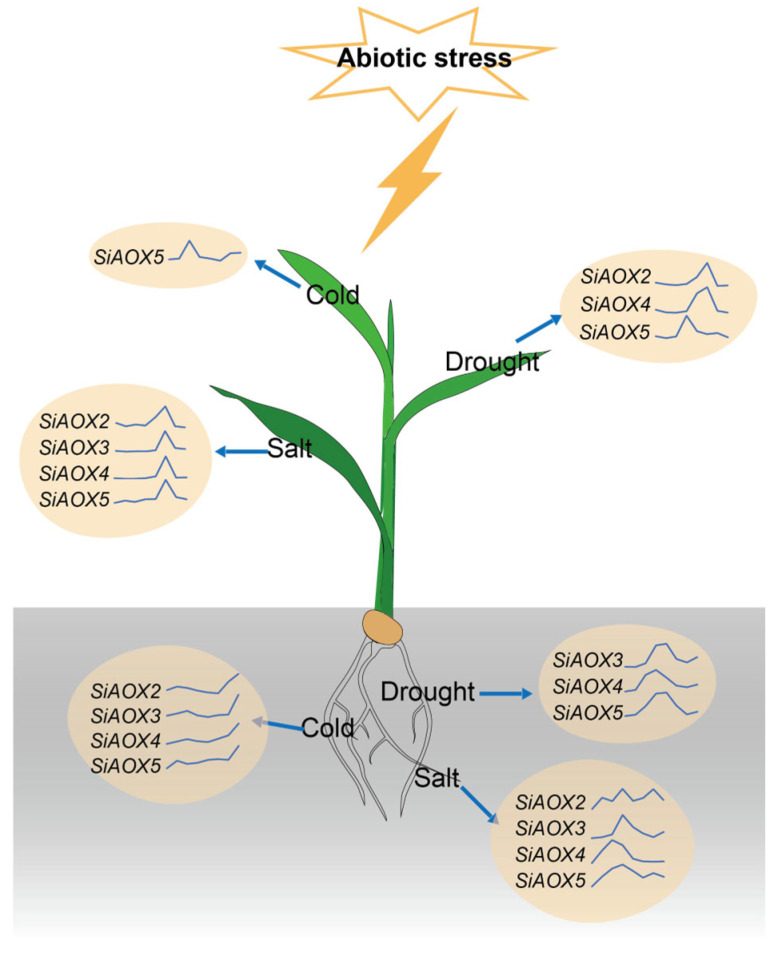
Model of the *SiAOX* genes responding to abiotic stress in foxtail millet.

In this study, we conducted a comprehensive genome-wide identification of *AOX* genes in foxtail millet, discovering a total of five *SiAOX* genes. The phylogenetic analysis systematically grouped these five SiAOX members into two subgroups: SiAOX1 was specifically assigned to the AOX1c clade, whereas SiAOX2–5 were coherently clustered within the AOX1d clade. We analyzed the basic characteristics, gene structures, three-dimensional configurations, conserved motifs, and *cis*-elements of these genes, thereby establishing a foundational understanding of the evolutionary relationships within the *SiAOX* gene family. Co-expression network analysis and examination of expression patterns for the *SiAOX* genes were carried out using RNA-seq and reverse transcription RT-qPCR. The results demonstrate that the five *SiAOX* genes exhibit tissue-specific expression and respond to abiotic stresses. Specifically, we identified four genes (*SiAOX2*, *SiAOX3*, *SiAOX4*, and *SiAOX5*) that are significantly upregulated in response to abiotic stresses, with distinct expression patterns observed between shoot and root tissues in foxtail millet seedlings (Figure 10). Haplotype analysis of *SiAOX4* and *SiAOX5* revealed that these genes are in linkage disequilibrium, with Hap_2 being the superior haplotype for both, potentially offering enhanced cold tolerance. These findings suggest that Hap_2 of both *SiAOX4* and *SiAOX5* may be targeted for selection in future breeding programs aimed at improving foxtail millet’s resilience to cold stress in a cultivar group.

## Figures and Tables

**Table 1:** Basic information and sequence characterizations of five *AOX* genes in foxtail millet.

Gene Name	Gene Structure		Protein				Subcelluar Localization
	CDS Length (bp)	Intron	AA Length	MW (kDa)	pI	GRAVY	
*SiAOX1*	1017	3	339	37.81	8.7	−0.138	Mitochondrial
*SiAOX2*	963	8	321	37.09	5.67	−0.239	Plasma Membrane
*SiAOX3*	1014	3	338	37.66	8.55	−0.151	Mitochondrial
*SiAOX4*	996	2	332	37.33	8.91	−0.283	Mitochondrial
*SiAOX5*	1014	2	338	37.21	7.31	−0.205	Mitochondrial

**Table 2:** *Ka*/*Ks* analysis for *SiAOX* family members.

Gene Name	Gene ID	*Ka*	*Ks*	*Ka*/*Ks*
*SiAOX1*	*Z._mays_AOX1c*	0.0684	0.3500	0.1954
*SiAOX1*	*S._bicolor_AOX1c*	0.0529	0.2425	0.2181
*SiAOX2*	*O._sativa_AOX1*	0.8616	1.8545	0.4646
*SiAOX3*	*O._sativa_AOX1d*	0.1832	0.4413	0.4153
*SiAOX4*	*Z._mays_AOX1d1*	0.0404	0.1853	0.2180
*SiAOX4*	*S._bicolor_AOX1d1*	0.0450	0.1687	0.2665
*SiAOX5*	*Z._mays_AOX1d2*	0.0433	0.2028	0.2134
*SiAOX5*	*S._bicolor_AOX1d2*	0.0344	0.2358	0.1460

## Data Availability

Data are contained within the article and Supplementary Materials.

## Supplementary Materials

The following supporting information can be downloaded at: https://www.mdpi.com/article/10.3390/plants13182565/s1, Figure S1: Distribution of *SiAOX* genes on chromosomes of foxtail millet. Table S1: Analysis of the secondary structure of SiAOX proteins. Table S2: Sequences of 60 AOX proteins from 17 species for phylogenetic analysis. Table S3: Predicted miRNAs targeting *SiAOX* family members. Table S4: Haplotype information of *SiAOX4*. Table S5: Haplotype information of *SiAOX5*. Table S6: Survive rate of *SiAOX4* after cold stress treatment. Table S7: Relative height of *SiAOX4* after cold stress treatment. Table S8: Survive rate of *SiAOX5* after cold stress treatment. Table S9: Relative height of *SiAOX5* after cold stress treatment. Table S10: Primer sequences using in RT-qPCR.

## References

[1] Pardo J., VanBuren R. (2021). Evolutionary innovations driving abiotic stress tolerance in C_4_ grasses and cereals. Plant Cell.

[2] Liang Y., Han Y. (2024). Pan-genome brings opportunities to revitalize the ancient crop foxtail millet. Plant Commun..

[3] Yang Z., Zhang H., Li X., Shen H., Gao J., Hou S., Zhang B., Mayes S., Bennett M., Ma J. (2020). A mini foxtail millet with an Arabidopsis-like life cycle as a C_4_ model system. Nat. Plants.

[4] Liang W., Liang H. (1997). Progress of the study on alternative oxidase. Chinese Bull. Bot..

[5] Florez-Sarasa I., Ribas-Carbo M., Del-Saz N.F., Schwahn K., Nikoloski Z., Fernie A.R., Flexas J. (2016). Unravelling the in vivo regulation and metabolic role of the alternative oxidase pathway in C_3_ species under photoinhibitory conditions. New Phytol..

[6] Zhang Y., Xu J., Li R., Ge Y., Li Y., Li R. (2023). Plants’ response to abiotic stress: Mechanisms and strategies. Int. J. Mol. Sci..

[7] Kim T.H., Bohmer M., Hu H., Nishimura N., Schroeder J.I. (2010). Guard cell signal transduction network: Advances in understanding abscisic acid, CO_2_, and Ca^2+^ signaling. Annu. Rev. Plant Biol..

[8] Cutler S.R., Rodriguez P.L., Finkelstein R.R., Abrams S.R. (2010). Abscisic acid: Emergence of a core signaling network. Annu. Rev. Plant Biol..

[9] Manan S., Zhao J. (2021). Role of *Glycine max ABSCISIC ACID INSENSITIVE 3* (*GmABI3*) in lipid biosynthesis and stress tolerance in soybean. Funct. Plant Biol..

[10] Manan S., Li P., Alfarraj S., Ansari M.J., Bilal M., Ullah M.W., Zhao J. (2024). FUS3: Orchestrating soybean plant development and boosting stress tolerance through metabolic pathway regulation. Plant Physiol. Biochem..

[11] Selinski J., Scheibe R., Day D.A., Whelan J. (2018). Alternative oxidase is positive for plant performance. Trends Plant Sci..

[12] Giraud E., Van Aken O., Ho L.H., Whelan J. (2009). The transcription factor *ABI4* is a regulator of mitochondrial retrograde expression of *ALTERNATIVE OXIDASE1a*. Plant physiol..

[13] Moore A.L., Siedow J.N. (1991). The regulation and nature of the cyanide-resistant alternative oxidase of plant mitochondria. Biochim. Biophys. Acta BBA—Bioenerg..

[14] Rhoads D.M., McIntosh L. (1991). Isolation and characterization of a cDNA clone encoding an alternative oxidase protein of *Sauromatum guttatum* (Schott). Proc. Natl. Acad. Sci. USA.

[15] Martin W., Rotte C., Hoffmeister M., Theissen U., Gelius-Dietrich G., Ahr S., Henze K. (2003). Early cell evolution, eukaryotes, anoxia, sulfide, oxygen, fungi first ?, and a tree of genomes revisited. IUBMB Life.

[16] Costa J.H., McDonald A.E., Arnholdt-Schmitt B., Fernandes de Melo D. (2014). A classification scheme for alternative oxidases reveals the taxonomic distribution and evolutionary history of the enzyme in angiosperms. Mitochondrion.

[17] Vanlerberghe G.C. (2013). Alternative oxidase: A mitochondrial respiratory pathway to maintain metabolic and signaling homeostasis during abiotic and biotic stress in plants. Int. J. Mol. Sci..

[18] Zhang D.W., Yuan S., Xu F., Zhu F., Yuan M., Ye H.X., Guo H.Q., Lv X., Yin Y., Lin H.H. (2016). Light intensity affects chlorophyll synthesis during greening process by metabolite signal from mitochondrial alternative oxidase in *Arabidopsis*. Plant Cell Environ..

[19] Demircan N., Cucun G., Uzilday B. (2020). Mitochondrial alternative oxidase (AOX1a) is required for the mitigation of arsenic-induced oxidative stress in *Arabidopsis thaliana*. Plant Biotechnol. Rep..

[20] Song C., Zhao Y., Li A., Qi S., Lin Q., Duan Y. (2021). Postharvest nitric oxide treatment induced the alternative oxidase pathway to enhance antioxidant capacity and chilling tolerance in peach fruit. Plant Physiol. Biochem..

[21] Hou L., Zhao M., Huang C., He Q., Zhang L., Zhang J. (2021). Alternative oxidase gene induced by nitric oxide is involved in the regulation of ROS and enhances the resistance of *Pleurotus ostreatus* to heat stress. Microb. Cell Fact..

[22] Zhu T., Zou L., Li Y., Yao X., Xu F., Deng X., Zhang D., Lin H. (2018). Mitochondrial alternative oxidase-dependent autophagy involved in ethylene-mediated drought tolerance in *Solanum lycopersicum*. Plant Biotechnol. J..

[23] Challabathula D., Analin B., Mohanan A., Bakka K. (2022). Differential modulation of photosynthesis, ROS and antioxidant enzyme activities in stress-sensitive and -tolerant rice cultivars during salinity and drought upon restriction of *COX* and *AOX* pathways of mitochondrial oxidative electron transport. J. Plant Physiol..

[24] Panda S.K., Sahoo L., Katsuhara M., Matsumoto H. (2013). Overexpression of alternative oxidase gene confers aluminum tolerance by altering the respiratory capacity and the response to oxidative stress in tobacco cells. Mol. Biotechnol..

[25] Garmash E.V., Velegzhaninov I.O., Ermolina K.V., Rybak A.V., Malyshev R.V. (2020). Altered levels of *AOX1a* expression result in changes in metabolic pathways in *Arabidopsis thaliana* plants acclimated to low dose rates of ultraviolet B radiation. Plant Sci..

[26] Grabelnych O.I., Borovik O.A., Tauson E.L., Pobezhimova T.P., Katyshev A.I., Pavlovskaya N.S., Koroleva N.A., Lyubushkina I.V., Bashmakov V.Y., Popov V.N. (2014). Mitochondrial energy-dissipating systems (alternative oxidase, uncoupling proteins, and external NADH dehydrogenase) are involved in development of frost-resistance of winter wheat seedlings. Biochemistry.

[27] Yang H., Deng L., Liu H., Fan S., Hua W., Liu J. (2019). Overexpression of *BnaAOX1b* confers tolerance to osmotic and salt stress in rapeseed. G3 Genes Genom. Genet..

[28] Oh G.G.K., O’Leary B.M., Signorelli S., Millar A.H. (2022). *Alternative oxidase* (*AOX*) *1a* and *1d* limit proline-induced oxidative stress and aid salinity recovery in *Arabidopsis*. Plant Physiol..

[29] Costa J.H., dos Santos C.P., e Lima B.d.S., Netto A.N.M., de Cruz Saraiva K.D., Arnholdt-Schmitt B. (2017). In silico identification of *alternative oxidase 2* (*AOX2*) in monocots: A new evolutionary scenario. J. Plant physiol..

[30] Vanlerberghe G.C., Dahal K., Alber N.A., Chadee A. (2020). Photosynthesis, respiration and growth: A carbon and energy balancing act for alternative oxidase. Mitochondrion.

[31] Borecký J., Nogueira F.T.S., de Oliveira K.A.P., Maia I.G., Vercesi A.E., Arruda P. (2006). The plant energy-dissipating mitochondrial systems: Depicting the genomic structure and the expression profiles of the gene families of uncoupling protein and alternative oxidase in monocots and dicots. J. Exp. Bot..

[32] Araújo Castro J., Gomes Ferreira M.D., Santana Silva R.J., Andrade B.S., Micheli F. (2017). *Alternative oxidase* (*AOX*) constitutes a small family of proteins in *Citrus clementina* and *Citrus sinensis* L. Osb. PLoS ONE.

[33] Wanniarachchi V.R., Dametto L., Sweetman C., Shavrukov Y., Day D.A., Jenkins C.L.D., Soole K.L. (2018). Alternative respiratory pathway component genes (*AOX* and *ND*) in rice and barley and their response to stress. Int. J. Mol. Sci..

[34] Ding C., Chen C., Su N., Lyu W., Yang J., Hu Z., Zhang M. (2021). Identification and characterization of a natural SNP variant in *ALTERNATIVE OXIDASE* gene associated with cold stress tolerance in watermelon. Plant Sci..

[35] Wang X., Geng X., Bi X., Li R., Chen Y., Lu C. (2022). Genome-wide identification of AOX family genes in Moso bamboo and functional analysis of *PeAOX1b_2* in drought and salinity stress tolerance. Plant Cell Rep..

[36] Zhang S., Yan C., Lu T., Fan Y., Ren Y., Zhao J., Shan X., Guan Y., Song P., Li D. (2023). New insights into molecular features of the genome-wide *AOX* family and their responses to various stresses in common wheat (*Triticum aestivum* L.). Gene.

[37] He Q., Wang C., He Q., Zhang J., Liang H., Lu Z., Xie K., Tang S., Zhou Y., Liu B. (2024). A complete reference genome assembly for foxtail millet and Setaria-db, a comprehensive database for *Setaria*. Mol. Plant.

[38] Qiao K., Yao X., Zhou Z., Xiong J., Fang K., Lan J., Xu F., Deng X., Zhang D., Lin H. (2023). Mitochondrial alternative oxidase enhanced ABA-mediated drought tolerance in *Solanum lycopersicum*. J. Plant Physiol..

[39] Zhang H., Wang Y., Zhao B., Zhang L., Qie Q., Han Y., Xukai L. (2023). Identification of co-expression genes related to cold stress in foxtail millet by WGCNA. J. Agric. Sci. Technol..

[40] Yokoyama R. (2023). AOX in action—Making plant mitochondrial metabolism special. Plant Physiol..

[41] Zhou S., Chen L., Chen H., Li Y., Chen G., Lu G., Yang H. (2022). Bioinformatics and expression analysis of *alternative oxidase* genes in sweetpotato. J. Nucl. Agric. Sci..

[42] Wang H., Ma Y., Qiao Z., Chang Y., Shu K., Ding H., Nie Y., Pan G. (2022). Structural and functional characterization of *AOX* gene family. Biotechnol. Bull..

[43] Millar A.H., Whelan J., Soole K.L., Day D.A. (2011). Organization and regulation of mitochondrial respiration in plants. Annu. Rev. Plant Biol..

[44] Mróz T.L., Havey M.J., Bartoszewski G. (2015). Cucumber possesses a single terminal alternative oxidase gene that is upregulated by cold stress and in the mosaic (MSC) mitochondrial mutants. Plant Mol. Biol. Rep..

[45] Costa J.H., Jolivet Y., Hasenfratz-Sauder M.P., Orellano E.G., da Guia Silva Lima M., Dizengremel P., Fernandes de Melo D. (2007). *Alternative oxidase* regulation in roots of *Vigna unguiculata* cultivars differing in drought/salt tolerance. J. Plant Physiol..

[46] Clifton R., Millar A.H., Whelan J. (2006). Alternative oxidases in Arabidopsis: A comparative analysis of differential expression in the gene family provides new insights into function of non-phosphorylating bypasses. Biochim. Biophys. Acta (BBA)—Bioenerg..

[47] Clifton R., Lister R., Parker K.L., Sappl P.G., Elhafez D., Millar A.H., Day D.A., Whelan J. (2005). Stress-induced co-expression of alternative respiratory chain components in *Arabidopsis thaliana*. Plant Mol. Biol..

[48] Watling J.R., Robinson S.A., Seymour R.S. (2006). Contribution of the alternative pathway to respiration during thermogenesis in flowers of the sacred lotus. Plant Physiol..

[49] Florez-Sarasa I., Lambers H., Wang X., Finnegan P.M., Ribas-Carbo M. (2014). The alternative respiratory pathway mediates carboxylate synthesis in white lupin cluster roots under phosphorus deprivation. Plant Cell Environ..

[50] Song X., Li Y., Cao X., Qi Y. (2019). MicroRNAs and their regulatory roles in plant-environment interactions. Annu. Rev. Plant Biol..

[51] Zhang L., Song J., Lin R., Tang M., Shao S., Yu J., Zhou Y. (2022). Tomato SlMYB15 transcription factor targeted by sly-miR156e-3p positively regulates ABA-mediated cold tolerance. J. Exp. Bot..

[52] Khandal H., Singh A.P., Chattopadhyay D. (2020). The MicroRNA397b-LACCASE2 module regulates root lignification under water and phosphate deficiency. Plant Physiol..

[53] Kang Y., Udvardi M. (2012). Global regulation of reactive oxygen species scavenging genes in alfalfa root and shoot under gradual drought stress and recovery. Plant Signal Behav..

[54] Mehta P.A., Rebala K.C., Venkataraman G., Parida A. (2009). A diurnally regulated dehydrin from *Avicennia marina* that shows nucleo-cytoplasmic localization and is phosphorylated by Casein kinase II in vitro. Plant Physiol. Biochem..

[55] Li X., Gao J., Song J., Guo K., Hou S., Wang X., He Q., Zhang Y., Zhang Y., Yang Y. (2022). Multi-omics analyses of 398 foxtail millet accessions reveal genomic regions associated with domestication, metabolite traits, and anti-inflammatory effects. Mol. Plant.

[56] Li X., Shi Z., Gao J., Wang X., Guo K. (2023). CandiHap: A haplotype analysis toolkit for natural variation study. Mol. Breed..

