# Supplementary material for: Genome-Wide Identification and Characterization of Alternative Oxidase (AOX) Genes in Foxtail Millet (Setaria italica): Insights into Their Abiotic Stress Response
Source: Plants (Basel). 2024 Sep 12;13(18):2565. doi: 10.3390/plants13182565 (PMC11434880; doi:10.3390/plants13182565)
Supplement: Supplementary file 1 [file plants-13-02565-s001.zip › Supplementary Figure S1, TableS1, S3, S10.pdf]

Supplementary materials:
Figure:

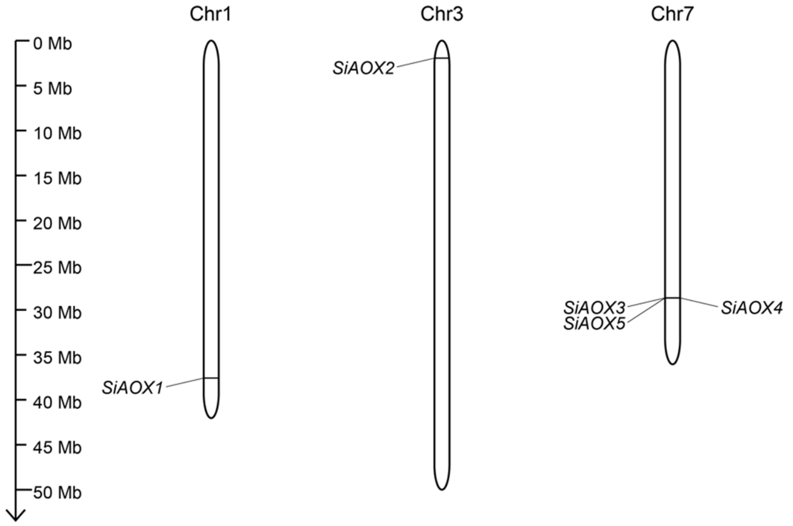

Figure S1. Distribution of *SiAOX* genes on chromosomes of foxtail millet.

Tables:

Table S1. Analysis of the secondary structure of SiAOX proteins

Name	Alpha helix	Extended strand	Beta turn	Random coil
SiAOX1	51.62	10.03	7.96	30.38
SiAOX2	54.21	6.23	4.36	35.20
SiAOX3	51.78	13.02	7.10	28.11
SiAOX4	51.81	7.83	3.61	36.75
SiAOX5	56.51	7.10	3.85	32.54

Table S3. Predicted miRNAs targeting *SiAOX* family members

miRNA accession	Target gene	Expectation	Target site	miRNA aligned fragment	Inhibition
sit-miR114-npr	*SiAOX1*	5.5	799-818	CUGAAGUGUUUGGGGAACUC	Cleavage
sit-miR148-npr	*SiAOX1*	6	835-855	AGUGGAUGGCGCGGGAGCUAA	Cleavage
sit-miR92-npr	*SiAOX1*	6	599-619	GGUGCUCGCUCUCUUCUGUCA	Cleavage
sit-miR33-npr	*SiAOX2*	4.5	302-322	UUACCAUGAUUGUAGAAUGUC	Cleavage
sit-miR101-npr	*SiAOX2*	5	434-453	GGCAGCUCUCCUCUGGCAGG	Cleavage
sit-miR13-npr	*SiAOX2*	5.5	474-495	UUGGAACUCGUGUUAGAAUUUC	Cleavage
sit-miR36-npr	*SiAOX2*	5.5	384-405	UGUGGAUCUAAAGUAUGGGAUG	Cleavage
sit-miR56-npr	*SiAOX2*	5.5	353-373	UCUGCAGCAUCGAAGAUCGCA	Translation
sit-miR162-npr	*SiAOX2*	6	353-373	ACUUCGUCGCAGAACGUCUUU	Cleavage
sit-miR2118c	*SiAOX2*	6	141-162	UUCCUGAUGCCUCUCAUUCCUA	Translation
sit-miR24-npr	*SiAOX2*	6	494-513	UUCAACUGGUUAGGAGGUGGG	Cleavage
sit-miR397	*SiAOX2*	6	309-329	UCAUUGAGUGCAGCGUUGAUG	Cleavage
sit-miR67-npr	*SiAOX2*	6	500-520	UCAAAUUUUGCAUGAUGAUAG	Translation
sit-miR38-npr	*SiAOX4*	4.5	122-142	UGGUGGUGGAAAUCGGCGCCA	Translation
sit-miR151-npr	*SiAOX4*	5.5	304-324	AGGGCUGAGGUUGAGCCACGU	Cleavage
sit-miR62-npr	*SiAOX4*	6	864-884	UCGAAGUUCAUGGACCUGGAU	Translation
sit-miR119-npr	*SiAOX5*	6	44-64	CGGUCGUGGGAUGACGCGGUA	Translation
sit-miR156-npr	*SiAOX5*	6	979-999	AGCGGGUUUAUCUGUGAGCCC	Translation

Table S10. Primer sequences using in RT-qPCR

Gene ID	Left primer (5'-3')	Right primer (5'-3')
SiACT	TGCTCAGTGGAGGCTCAACA	CCAGACACTGTACTTGCGCTC
SiAOX1	GTCGTTACCGTGGTGAGGGC	TCCTTGAGCTCCATCCCCTG
SiAOX2	AGAAGCTGATACGGAAGGTG	CAGGTCTCGGTCTTTACTTT
SiAOX3	CCCGCCAACGCCACGCTCAA	TGGATGTCCGATGCGAAGTG
SiAOX4	AGGGGATGAAGCTGAAGGAC	CGAGGGGTAGCAGGTAATGT
SiAOX5	CGACGGCACGGAGTGGAAGT	CCGGCGCTGGAAGAAGAGGT
